# Clinical Consensus Statement: Perioperative Management of Prothrombotics, Antiplatelets, Anticoagulants, DMARDs and Biologics in Elective Podiatric Surgery

**DOI:** 10.1002/jfa2.70121

**Published:** 2026-03-23

**Authors:** Jade Tang, Lesley Posmyk, James Cowden, Richard Lang, Helen Milnes, Lorna Donson

**Affiliations:** ^1^ Braintree Rehabilitation Hospital: Encompass Health Rehabilitation Hospital of Braintree Chelmsford UK; ^2^ North Tees and Hartlepool NHS Foundation Trust Stockton‐on‐Tees UK; ^3^ Nottinghamshire Healthcare NHS Foundation Trust Nottingham UK; ^4^ Sheffield Teaching Hospitals NHS Foundation Trust Sheffield UK; ^5^ Essex Partnership University NHS Foundation Trust Essex UK; ^6^ East Birmingham Hospital: Birmingham Heartlands Hospital Birmingham UK; ^7^ Wye Valley NHS Trust Hereford UK

**Keywords:** anticoagulants, antiplatelets, biologics, disease‐modifying anti‐rheumatic drugs, foot surgery, prothrombotics

## Abstract

**Introduction:**

A substantial body of literature exists on the perioperative management of pharmacological agents specifically prothrombotic agents, antiplatelets, anticoagulants and disease‐modifying antirheumatic drugs (DMARDs) aimed at mitigating risks associated with both the medications themselves and the comorbidities they treat. However, a clear and consistent consensus on best practice is either lacking or poorly defined especially in foot surgery. To address this gap, a working group of podiatric surgeons convened to develop a Clinical Consensus Statement (CCS) for the perioperative management of these drugs in foot and ankle surgery.

**Method:**

A five‐member panel identified four key areas of interest: prothrombotic agents, antiplatelets, anticoagulants and disease‐modifying antirheumatic drugs. They conducted a comprehensive literature review to assess the available data and identify evidence gaps, ultimately formulating nineteen questions grouped within these areas using a modified Delphi method. These questions were presented to delegates at the 2022 Faculty of Podiatric Surgery (FoPS) Conference.

**Results:**

The recommendations from the clinical consensus statement are summarised in Appendix 1 highlighting the strength of each recommendation. Although more high‐level evidence is needed to inform decision‐making in this area, this consensus statement has utilised the available evidence base and reflects current practice. Rather than providing a definitive guideline, it provides valuable interim guidance for elective foot surgery, however, does not replace local trust policies and guidelines.

## 1 Introduction

This document was developed as a CCS for the Royal College of Podiatry Faculty of Podiatric Surgery. It is important to clarify that this CCS does not constitute clinical guidelines, formal NHS trust policies, comprehensive evidence reviews or official recommendations. Instead, it reflects the collective knowledge and insights of a panel of experts based on the best available evidence. It may also include differing viewpoints, uncertainties and minority opinions. The primary aim of a CCS is to stimulate discussion rather than offer definitive solutions. Treatment outcomes are not guaranteed by following a CCS, as final decisions rest with the treating healthcare professional (HCP), who should tailor treatment according to clinical information, specific circumstances and individual patient need.

With an ageing population and the increasing prevalence of comorbidities and complex polypharmacy in the perioperative setting, the impact of medications on intraoperative and postoperative outcomes must be carefully assessed prior to surgery to minimise complications and optimise patient outcomes. Key medications of concern that influence podiatric surgeons' perioperative decision‐making include prothrombotic agents, antiplatelets, anticoagulants and disease‐modifying antirheumatic drugs. Evidence supporting best practices in these pharmacological areas is generally limited and inconclusive especially in the context of elective foot and ankle surgery.

This CCS addresses the challenges related to managing these medications, such as whether to continue, interrupt or bridge treatment as well as the duration of interruption and collaboration with other HCPs. As with any medical intervention, the risks and benefits of continuing or discontinuing a medication must be carefully weighed. Factors to consider include intraoperative complications and postoperative adverse effects, such as pain, swelling, infection and excessive bleeding.

## 2 Methodology

### 2.1 Panel and Development of Questions

A five‐member panel, consisting of four Podiatric Surgeons and a Consultant Anaesthetist, was established to identify areas of interest and evidence gaps in the perioperative management of pharmacology in foot and ankle surgery. The panel created questions aiming to support consensus on pharmacological management in podiatric surgery, focussing on the following identified areas of interest, prothrombotics, antiplatelets, anticoagulants and disease‐modifying antirheumatic drugs.

The panel conducted a preliminary literature review of current research on the four areas of interest using databases such as MEDLINE, CINAHL, ScienceDirect and Elsevier. Following this search, they compiled a series of questions with multiple choice answers for each area to establish consensus on management strategies in podiatric surgery and address known variations in clinical practices.

### 2.2 Study Design

The Delphi method was adapted to capture high engagement at the conference rather than remote multiround responses. Nineteen questions divided into the four topics of interest (Appendix 1: Table A1). Participants responded anonymously via Slido, an interactive smartphone application. The consensus levels were predetermined (Table 1) with the study aiming to achieve a strong consensus of 75% or more for each question.

**TABLE 1:** Consensus levels.

Consensus outcome	Consensus percentage
High	> 75%
Moderate	65%–74%
Low	56%–64%
Consensus not met	< 55%

### 2.3 Participants and Data Collection

Delegates at the 2022 Faculty of Podiatric Surgery conference were used as a convenience sample, providing a larger response rate with specialised knowledge of foot and ankle surgery, thereby enhancing the relevance and expertise of the consensus. Participants were informed that their responses would contribute to the consensus statements, and consent was implied through their participation. Responses to the questions were collected using the Slido application. Responses were reviewed and compiled to assess whether consensus was reached for each question and to determine the strength of that consensus.

### 2.4 Surgery Classification

To ensure consistency and relevance in the responses, surgical procedures were categorised into three distinct classes (Table 2). This provided a structured framework when answering the questions.

**TABLE 2:** Classification of surgery.

Class 1	Cutaneous surgery, nail surgery, needling, curettage
Class 2	Digital procedures, subcutaneous surgery e.g., ganglion excision, neurectomy, minor amputation, osteotomies or fusions without cast immobilisation
Class 3	Osteotomies or fusions with cast immobilisation

### 2.5 Prothrombotics

#### 2.5.1 Question 1: Should We Advise Patients to Stop Taking Oral Oestrogen‐Based HRT/OCP Prior to Surgery?

Consensus statement: Responses to this question were lost and could not be recollected; published guidance is referenced instead. The Department of Health (DoH, 2010) has published a risk assessment for venous thromboembolism (VTE), identifying hormone replacement therapy (HRT) as a significant risk factor that may warrant thrombo‐prophylaxis during the perioperative period [1]. Similarly, current NICE guidance (NG89, 2018) recommends considering discontinuation of oestrogen‐containing contraceptive pills (OCP)/HRT 4 weeks prior to major surgery [1].

The association between oral oestrogen‐containing compounds and VTE has been extensively studied. The overall risk of developing VTE is influenced by factors such as treatment duration, the generation of the medication, dosage and formulation of oestrogen products [2, 3, 4].

A systematic review of VTE prophylaxis for foot and ankle surgery reported a low VTE risk of 0%–0.55%, identifying the contraceptive pill as a contributing factor [5]. Similarly, a meta‐analysis on VTE risk and prophylaxis in foot and ankle surgery found an incidence of VTE without prophylaxis below 1% with one study specifically citing HRT as a notable risk factor [6]. Felcher et al. (2009) [7] observed a 0.55% increased risk of VTE in patients undergoing foot and ankle surgery who were taking HRT/OCP and determined it as a significant predictive factor [8]. Evidence remains inconclusive. Although some studies suggest a low overall VTE risk in foot and ankle surgery, the argument for stopping HRT/OCP in the perioperative period is still debated, indicating further research is required [5, 6].

#### 2.5.2 Question 2: At What Point Should We Advise Patients to Stop Oestrogen‐Based HRT/OCP Prior to Surgery?

Consensus statement: A high consensus was reached (97%) recommending stopping oestrogen‐based medication 4 weeks before elective surgery with 3% supporting cessation at 2 weeks. This aligns with national guidance (NICE, NG89, 2018, updated 2019), which recommends cessation between 2 and 4 weeks, with a minimum of 4 weeks before major surgery [2, 9]. Studies show plasma concentrations of antithrombin III rebound within 2–6 weeks after OCP cessation with most changes in coagulation activity occurring in the first 1–2 weeks [10, 11]. However, the Robinson et al. Study (1991), which informed national recommendations, has been criticised for its low quality and lack of assessment of certain coagulation markers [12].

#### 2.5.3 Question 3: If a Patient Were to Refuse to Interrupt Their Oral Oestrogen‐Based HRT/OCP Should We?

Consensus statement: a high consensus (100%) was reached to proceed with surgery with the addition of VTE chemoprophylaxis if a patient refuses to discontinue their oral oestrogen‐based HRT or OCP.

When understanding a patient's decision to continue HRT or OCP, it is important to consider the various factors involved. These medications are used not only for contraception but also for treating dysmenorrhoea, abnormal uterine bleeding, acne, hirsutism, endometriosis pain, gender transition and reducing the risk of ovarian and endometrial cancers [13, 14]. Concerns related to fertility, menstruation and the risk of side effects may influence the patient's decision and affect the pre‐ and post‐operative period [15, 16, 17]. Sudden oestrogen withdrawal can lead to symptoms such as hot flushes, night sweats and vasomotor instability [18]. These factors must be carefully weighed and respected when managing patients who choose to continue HRT or OCP.

#### 2.5.4 Question 4: When Should We Advise Patients to Restart Their Oestrogen‐Based HRT/OCP?

Consensus statement: 99% recommended restarting HRT/OCP once patients begin mobilising after surgery. Pro‐thrombotic clotting factor changes can persist for 4–6 weeks after hormone discontinuation, whereas the effects of hormone therapy may take several months to re‐establish after re‐initiation. Foot surgery patients mobilise early and therefore the overall risk is low, while resuming oestrogen therapy helps avoid prolonged oestrogen deficiency [10, 17].

In podiatric surgery, most procedures are day cases, allowing for same‐day ambulation. Weight‐bearing activity activates the calf muscle pump, reducing VTE risk, which is largely due to reduced calf pump activity during immobilisation [19, 20]. For low‐risk VTE procedures involving the foot and ankle, early mobilisation ensures the calf pump is effective, making it a suitable time to restart the patient's HRT/OCP.

### 2.6 Antiplatelets

#### 2.6.1 Question 1: Should We Routinely Interrupt Aspirin Preoperatively?

Consensus statement: A high consensus (96%) advised against routinely stopping aspirin preoperatively with 4% recommending discontinuation for class 2 and 3 surgeries (1%) or class 3 surgeries only (2%). American College of Cardiology/American Heart Association Joint Committee (2024) guidance recommends the continuation of perioperative aspirin in patients with cardiovascular disease who are undergoing elective orthopaedic surgery with a low bleed risk: this consensus aligns with those guidelines and other authors [21, 22, 23, 24]. High consensus (96%) advised against routinely stopping aspirin preoperatively with 4% recommending discontinuation for class 2 and 3 surgeries (1%) or class 3 surgeries only (2%). American College of Cardiology/American Heart Association Joint Committee (2024) guidance recommends the continuation of perioperative aspirin in patients with cardiovascular disease who are undergoing elective orthopaedic surgery with a low bleed risk: this consensus aligns with those guidelines and other authors [21, 22, 23, 24].

The decision to stop or continue aspirin before noncardiac surgery (NCS) requires balancing bleeding and thrombotic risks. Aspirin increases surgical bleeding, but discontinuation raises the risk of thrombotic events especially in patients with cardiovascular disease or recent coronary stents [25, 26]. Surgery induces a hypercoagulable state, increasing the likelihood of thrombosis and the severe consequences of stopping antiplatelet therapy (APT) perioperatively particularly in cardiovascular patients [21]. A meta‐analysis shows that while continuing aspirin raises post‐operative bleeding risk by 1.5 times, the severity of bleeds generally remains unaffected (except in neurosurgery and prostatectomy) [27]. However, Plümer et al. (2017) [28] observed that stopping aspirin used for secondary prevention can increase the risk of myocardial infarction by up to 60% relative to continuation. It is therefore recommended to continue aspirin perioperatively for elective podiatric surgery with low bleeding risk. Decisions should be individualised to carefully balance the risks of bleeding and thrombosis: Table 3 provides a summary of considerations for this risk balance.

**TABLE 3:** Summary of considerations for stopping aspirin perioperatively.

Perioperative bleeding risk	Blood transfusion requirement	Type of procedure
Low	Usually not required	Peripheral, plastic, and general surgery biopsies
Easily achieved local haemostasis		Minor orthopaedic and general surgery
		Endoscopic procedures
		Carotid and peripheral vascular surgery
		Dental extraction and surgery
		Breast surgery
Intermediate	May be required	Majora intrathoracic and intraperitoneal surgery
Potentially difficult to achieve local haemostasis; risk of reintervention		Cardiac surgery
		Open aorta surgery
		Major orthopaedic surgery
		Urological surgery
		Reconstructive surgery
High	Usually required	Intracranial neurosurgery
Difficult to achieve local haemostasis; possible bleeding in a closed space; impact on mortality and surgery outcome		Extensive thoracoabdominal dissection

*Source:* [21].

#### 2.6.2 Question 2: If We Stop Aspirin Routinely, When Should We Do So?

Consensus Statement: A high consensus (91%) indicated that this question was not applicable. An outlier (1%) stated they would omit aspirin on the day of surgery, while 8% suggested stopping it 7 days preoperatively.

It is generally accepted that aspirin should be continued for elective patients whenever possible [29]. However, discontinuing aspirin may be beneficial for patients with lower cardiovascular risk, such as those on primary prevention (no previous history of event), who are undergoing higher bleeding risk procedures [27]. If aspirin is to be interrupted preoperatively, guidelines recommend discontinuation five to 7 days prior to the procedure [21, 30]. This timeframe allows the antiplatelet effects of aspirin to diminish (based on the lifespan of platelets), thereby reducing the risk of excessive bleeding during surgery.

#### 2.6.3 Question 3: When Should We Restart Aspirin?

Consensus statement: A high consensus of 96% indicated that restarting aspirin was not applicable, while 4% recommended resuming it the day after surgery. Discussions between surgical and cardiovascular teams are crucial to determine the optimal treatment based on the patient's risk profile, including the severity of cardiovascular disease, surgery type and bleeding risk.

Aspirin should be continued whenever possible as part of secondary prevention therapy. If interrupted, research suggests resuming aspirin as soon as feasible, ideally on the same day as the intervention, when the risk of ischaemic complications is highest in patients with high cardiovascular risk [21]. Perioperative withdrawal for over 8 days significantly increases the rates of major adverse cardiovascular events (MACE) [25, 29].

For surgeries with a high bleeding risk, a more cautious approach may be warranted with aspirin potentially restarted 48–72 h post‐operatively, or later, based on the surgeon's assessment of bleeding risk and the patient's condition [30]. Monitoring may be essential when restarting aspirin within the first 48 h post‐operatively, as this can increase bleeding risk and potentially affect healing. Table 4 summarises the recommendations for aspirin interruption [30].

**TABLE 4:** Summary of perioperative aspirin interruption and restart timing [30].

Minor surgery
Do not stop antiplatelet therapy
Implement multidisciplinary consult in patient with (potential) bleeding complications. Low molecular weight heparin: NOT a substitute for platelet inhibiting drugs.
Avoid plasmic anticoagulation (LMWH) during surgery.

**Major Survey**	**How to proceed**	**Exception**	**How to proceed with exception**
Aspirin for primary prevention^a^	Stop aspirin for 5 days before surgery^a^		
Aspirin in high risk patients^a^ (Diabetes, history of cardiovascular events, known cardiovascular disease, increased global risk)	Continue aspirin^a^	Surgery in close space, expected major bleeding complications	Stop aspirin 5 days before surgery^a^ Consider restarting within 24 h^a^
Aspirin *plus* clopidogrel in high risk patients	Elective surgery: Delay until no dual inhibition necessary Semi‐urgent surgery: Continue aspirin +/− clopidogrel on a case by case basis Urgent surgery (within 24 h): Continue aspirin and clopidogrel	Surgery in closed space, expected major bleeding complications	If delaying surgery is not possible/semi‐urgent surgery is necessary: Stop clopidogrel 5 days before surgery, consider bridging with short activing GPIIb/IIIa antagonist Consider also stopping aspirin in particular patients Consider resuming dual antiplatelet therapy as soon as possible

^a^ Applies to patients also on clopidogrel monotherapy.

#### 2.6.4 Question 4: If a Patient Is on Dual Antiplatelet Therapy (DAPT), Should We

- Interrupt neither
- Seek specialist opinion
- Wait 12 months until dual therapy ceases
- Liaise with GP to determine why they remain on dual therapy

Consensus Statement: A moderate consensus (67%) indicated that seeking specialist opinion for preoperative DAPT management is preferred. Meanwhile, 18% recommended delaying surgery for 12 months, and 15% suggested consulting the patient's general practitioner. Guidelines recommend seeking specialist advice for preoperative DAPT management due to the complexities and risks involved in balancing bleeding and thrombosis [31, 32]. Early interruption of DAPT is associated with a higher risk of death and re‐hospitalisation [33] consulting with specialists ensure individually tailored care, helping to identify the safest course of action for each patient [21, 25].

Research recommends continuing DAPT for the full prescribed duration to minimise the risk of major adverse cardiovascular events particularly for procedures that can be postponed or have a very low bleeding risk [21, 29, 31]. The risk of major events is highest within the first month following coronary intervention and gradually decreases over time [31]. The 2014 guidelines from the American College of Cardiology and the American Heart Association recommended delaying elective noncardiac surgery for one year. These guidelines were updated in 2016, reducing the recommended waiting period to six months [22, 23, 24].

#### 2.6.5 Question 5: Should We Stop Clopidogrel and ‘Bridge’ With Aspirin?

Consensus statement: A low consensus (59%) indicated that the group would not discontinue clopidogrel preoperatively, 28% would stop clopidogrel and bridge with aspirin, whereas 13% would stop without bridging.

The primary concern with perioperative management of clopidogrel is balancing the risk of thrombosis against bleeding during surgery. Although some guidelines suggest continuing clopidogrel monotherapy in similar cases as aspirin [34], evidence remains insufficient to make a definitive recommendation [29, 33].

Interestingly, a recent meta‐analysis found no statistically significant difference in the risk of major bleeding or thromboembolism between stopping and continuing APT perioperatively [33]. Furthermore, a systematic review found no prospective studies with the claim that continuing clopidogrel increases bleeding risk based only on indirect retrospective evidence [32].

Although some guidelines suggest continuing clopidogrel in cases such as aspirin, the evidence is insufficient to make a clear recommendation [29]. Furthermore, the advice to switch from clopidogrel to aspirin before surgery is mixed and inconsistent [32, 33]. Notably, current BNF guidelines recommend stopping clopidogrel 7 days before elective surgery if its antiplatelet effects are not desired [35]. Given the lack of consensus and limited data on clopidogrel monotherapy, decisions should involve a multidisciplinary approach considering a patient's individual risk profile.

### 2.7 Anticoagulants

#### 2.7.1 Question 1: Should We Routinely Stop Direct Oral Anticoagulants (DOACS) Preoperatively?

Consensus Statement: A strong consensus (92%) agreed that DOACs should be interrupted before surgery; however, there was variation in the types of surgery to which this applied. The majority (59%) indicated they would stop DOACs for all surgeries, whereas 27% stated they would only pause them for class 2 and 3 surgeries, and 6% indicated they would only stop for class 3 surgeries.

Guidance recommends that the decision to stop DOACs should consider the estimated thromboembolic risk associated with discontinuation and the bleeding risk associated with continuation [36]. The duration of anticoagulant discontinuation should be minimised to reduce thromboembolic risk. Steffel et al. (2021) [37] suggest that small orthopaedic procedures, including those involving the foot, are considered low‐risk interventions, indicating that it may be appropriate to stop anticoagulants in this patient cohort.

#### 2.7.2 Question 2: When Should We Stop DOACs Preoperatively?

Consensus Statement: A high consensus (76%) agreed that local trust guidance should be followed, whereas 24% would typically stop DOACs 48 h before surgery if eGFR is normal. The main advantage of DOACs is their rapid onset and short half‐life. Literature suggests DOAC interruption should be based on their half‐lives with four to five elimination half‐lives considered an appropriate timescale [38, 39].

The 2019 PAUSE study found low rates of major bleeding and thromboembolism when direct factor Xa inhibitors were stopped one day before low‐risk procedures and 2 days before high‐risk procedures. This is reflected in several guidelines, including those from the British Society for Haematology (2016, updated 2022) [40].

DOAC interruption may require adjustment in cases of renal or severe hepatic impairment as well as for patients taking drugs that reduce DOAC clearance or are primarily metabolised by the kidneys [41, 42]. Additionally, due to the increased risk associated with neuraxial blocks, direct Factor Xa inhibitors should be stopped 72 h before these interventions [43].

#### 2.7.3 Question 3: When Should We Restart DOACS Post‐Operatively?

Consensus Statement: A high consensus (83%) agreed that DOACs should be restarted the day after surgery, whereas 9% recommended waiting 2 days post‐surgery. The remaining 5% preferred restarting on the same day as the procedure.

Due to their rapid onset of action with peak effects occurring one to 3 hours post‐intake, caution is advised when restarting DOACs after surgery [38]. The British Society for Haematology guidelines (2016, updated 2022) [40] recommend resuming DOACs similarly to their cessation: one day post‐operatively for low‐bleeding‐risk procedures and two to three days post‐operatively for high‐bleeding‐risk procedures.

### 2.8 Disease‐Modifying Antirheumatic Drugs (DMARDs)

#### 2.8.1 Question 1: Should We Routinely Interrupt DMARDs Perioperatively?

Consensus Statement: A high consensus (97%) supports continuing DMARDs perioperatively with only 3% recommending discontinuation. Decisions on interrupting DMARDs during podiatric surgery involve balancing infection risk with potential disease flare‐ups, considering factors such as the type of surgery, the patient's condition and the specific DMARD used.

Studies indicate that continuing conventional DMARDs in rheumatoid patients undergoing elective orthopaedic surgery does not increase post‐operative infection risk or delay wound healing with most research favouring perioperative continuation [44, 45]. Randomised controlled trials also show that patients who continued methotrexate (MTX) had lower complication rates (2% vs 15%) and no disease reactivation 6 months post‐surgery compared to 8% in those who discontinued MTX [46]. Research further supports MTX continuation to prevent disease reactivation without added complication risk [47, 48].

#### 2.8.2 Question 2: Should We Decide When the Patient Stops DMARDS?

Consensus statement: A high consensus (75%) agreed that DMARDs should not be interrupted perioperatively. The remaining 25% recommended seeking specialist guidance or following local trust guidelines.

Continuing conventional DMARDs is often preferred especially for low‐risk surgeries such as minor podiatric procedures, as discontinuation may provoke a flare without significantly reducing infection risk. The risk level of surgery is essential in decision‐making for patients on DMARDs. Balancing infection risk against the potential for disease flare requires individualised decisions and careful perioperative planning.

When evaluating whether to interrupt DMARD therapy, a multidisciplinary team approach is essential. Collaboration between rheumatologists and surgeons enables a personalised plan that considers the patient's overall health, DMARD type and specific surgical factors, ensuring thorough assessment. Key patient risk factors include steroid use, diabetes and hypertension, whereas advanced age and longer surgery duration have been identified as infection risks [44]. Additionally, rheumatoid arthritis itself may increase the likelihood of surgical complications [49].

#### 2.8.3 Question 3: When Should DMARDs Be Restarted Post‐Operatively?

Consensus Statement: A high consensus (90%) agreed that DMARDs should not be interrupted perioperatively. Of the remaining respondents, 8% preferred resuming DMARDs once the surgical wound has healed, and 1% indicated they would seek specialist guidance. Overall, the consensus supports the continuation of conventional DMARD therapy during the perioperative period without significantly increasing the risk of infection or compromising wound healing. Franco et al. (2017) noted that most prospective studies favour continuing conventional DMARDs during this period.

There is no universal guideline but wound healing issues are more common in patients with rheumatoid arthritis than those without and so patient‐specific factors such as overall health, immune status and comorbidities such as diabetes, and steroid use may need to be considered when tailoring DMARD management [44].

Research indicates that, although foot surgery does not increase the risk of post‐operative surgical site infections (SSIs), factors such as preoperative deformities and specific surgical techniques can lead to delayed wound healing. Notably, post‐operative swelling in the forefoot and dorsal foot is more severe, particularly in patients with rheumatoid arthritis, necessitating close monitoring [50].

### 2.9 Biologics

#### 2.9.1 Question 1: Should We Interrupt Biologics Perioperatively?

Consensus statement: Low consensus (59%) favoured scheduling surgery at a specific treatment time. The remaining 21% would decide based on patient condition and risk, 13% would seek specialist input and 6% would routinely interrupt biologics.

Anti‐tumour necrosis factor (anti‐TNF) medications have been linked to increased risks of SSI and VTE [51, 52]. In contrast, den Broder et al. (2007) found no increased risk of SSI but did report higher rates of wound dehiscence and postoperative bleeding in patients who continued anti‐TNF therapy.

Although high‐quality evidence is limited, existing literature and guidelines support stopping biologic therapies preoperatively for most procedures except for bloodless ones such as cataract surgery or biopsy. As with other medications discussed in this paper, the risks of continuing biologics should be weighed against the potential for disease flare if the medication is discontinued.

#### 2.9.2 Question 2: Should Our Decision to Interrupt Biologics Be Dependent on the Procedure Being Undertaken?

Consensus statement: The group did not reach consensus on whether to interrupt biologics based on procedure type: 43% voted ‘no,’ 1% supported interruption for Class 3 surgeries only, 38% for Classes 2% and 3% and 18% for all classes. However, the current literature and guidance advise stopping for all procedures, but duration of pre‐op cessation is determined by the inherent bleeding risk of the procedure.

#### 2.9.3 Question 3: When Should We Aim to Operate on a Patient Receiving Biologic Therapy?

Consensus statement: A moderate consensus was reached with 74% voting to operate at the end of a patient's drug cycle, 23% preferring to consult with the medical team overseeing the biologic regimen and the remaining 3% opting to operate mid‐cycle.

It is recognised that SSI risk varies depending on surgical procedure with some procedures, such as major orthopaedic surgery, carrying a higher risk. The British Society of Rheumatology (2019) guidelines recommend allowing one dose interval to elapse before most procedures with surgery scheduled at least 1 week after the next dose would be due. For higher risk procedures, however, the guidelines advise stopping biologics for 3–5 times the drug's half‐life before surgery. Rituximab is recommended to be stopped 3–6 months prior to elective surgery. A full list of time intervals prior to surgery has been provided in Table 5.

**TABLE 5:** Dosing intervals and recommendations for timing of surgery of biologic medications.

Drug	Dosing interval	Period in which surgery should be scheduled (relative to last biologic dose administered)	One half‐life, days	Five half‐lives, days
Adalimumab	Every 2 weeks	Week 3	14	70
Abatacept IV/SC	Monthly (IV)	Week 5	14	70
	Weekly (SC)	Week 2		
Certolizumab	Every 2 weeks	Week 3	14	70
	Every 4 weeks	Week 5		
Etanercept	Weekly or twice weekly	Week 2	3	15
Golimumab	Every 4 weeks	Week 5	14	70
Infliximab	Every 4, 6, or 8 weeks	Week 5, 7 or 9	9	45
Rituximab	Two doses 2 weeks apart, no more frequent than every 6 months	Months 4–7	18	90
Tocilizumab IV	Every 4 weeks	Week 5	11	55
4 mg/kg				
8 mg/kg			13	65
Tocilizumab SC	Every week	Week 3	13	65
Ustekinumab	Every 12 weeks	Week 13	21	105

*Note:* (British Society of Rheumatology Guidelines, 2019).

It is considered good practice to consult with the individual's rheumatologist to ensure the most appropriate plan, considering personal factors such as infection history and the stability of their inflammatory disease.

#### 2.9.4 Question 3: When Should We Consider Recommencement of Biologic Treatment?

Consensus statement: A high consensus was reached with 85% voting to recommence biologic therapy once the surgical wound has fully healed. Another 11% preferred seeking a specialist opinion before restarting therapy, whereas 5% felt that the treatment regimen should remain uninterrupted.

This consensus decision aligns with British Rheumatology guidelines, which recommends that biologic therapy not be resumed until wound healing is achieved (typically around 14 days) and there are no signs of infection [53].

## 3 Conclusion

This clinical consensus statement has aimed to provide an overview of the current literature and guidance recommendations pertaining to the perioperative management of prothrombotics, anticoagulants, antiplatelets, DMARDs and biologics in elective podiatric surgery. The experiences of delegates provided valuable broad professional insight that said the limitations of a convenience sample is recognised. A multidisciplinary approach to perioperative management in elective foot and ankle surgery is strongly recommended. It is imperative to remember that this is a consensus document only and demonstrates the consensus of a specialist group but does not constitute national guidelines nor replace local Trust policies and guidelines.

Although one question's responses were lost, all other data were verified, and findings remain robust. *This process has also highlighted specific gaps in the current evidence base, underlining the need for further high‐quality research to inform future guidance*.

## Author Contributions

**Jade Tang:** conceptualization, data curation, formal analysis, methodology, project administration, writing – original draft, writing – review and editing. **Lesley Posmyk:** conceptualization, data curation, formal analysis, methodology, project administration, writing – original draft, writing – review and editing. **James Cowden:** conceptualization, data curation, formal analysis, methodology, writing – original draft. **Richard Lang:** conceptualization, data curation, formal analysis, methodology, writing – original draft. **Helen Milnes:** conceptualization, data curation, formal analysis, methodology, writing – original draft. **Lorna Donson:** conceptualization, data curation, formal analysis, methodology, writing – original draft.

## Funding

The authors have nothing to report.

## Ethics Statement

The authors have nothing to report.

## Consent

The authors have nothing to report.

## Conflicts of Interest

The authors declare no conflicts of interest.

## Data Availability

The data that support the findings of this study are available from the corresponding author upon reasonable request.

## Appendix

**TABLE A1:** Table of questions and consensus voting scores.

Question	Multiple choice answers	Consensus reached% (*n* = )
Prothrombotics		
1. Should we advise patients to stop taking oral oestrogen‐based HRT/OCP prior to surgery?	No	Data not provided to authors
	Yes, all patients	
	Yes, class 2 and 3	
	Yes, only class 3	
	Unsure	
2. At what point should we advise patients to stop oestrogen‐based HRT/OCP prior to surgery?	Not applicable, we should not interrupt oral oestrogen‐ based HRT/OCP	0% 0/74
	4 weeks	97% 72/74
	Other, please state:	3% 2/74
3. If a patient were to refuse to interrupt their oral oestrogen‐based HRT/OCP should we?	Refuse to proceed with surgery	0% 0/55
	Proceed to surgery with consideration of chemical prophylaxis	100% 55/55***
4. When should we advise patients to restart their oral oestrogen‐based HRT/OCP?	Not applicable, we should not operate	0% 0/70
	Day after surgery	1% 1/70
	Once mobile i.e. calf pump active	99% 69/70***
Antiplatelets		
1. Should we routinely interrupt aspirin therapy preoperatively?	No	96% 8/71***
	Yes, for all classes	0% 0/71
	Yes, class 2 and 3	1% 1/7
	Yes, only class 3	3% 2/71
2. If you stop aspirin routinely, when should we stop it?	Not applicable, should not stop aspirin	91% 69/76***
	Omit on the day of surgery	1% 1/76
	7 days preoperatively	8% 6/76
3. When should we restart aspirin?	Not applicable, should not stop aspirin anyway	96% 69/72***
	The day after surgery	4% 3/72
4. If a patient is on dual antiplatelet therapy, should we:	Interrupt neither	0% 0/78
	Seek specialist opinion	67% 52/78**
	Wait 12 months until dual therapy ceases	18% 14/78
	Liaise with GP to determine why they remain on dual therapy	15% 12/78
5. Should we stop clopidogrel and ‘bridge’ with aspirin?	No, we should not stop clopidogrel	59%*
	No, we should not bridge, just stop it	13%
	Yes, I would bridge with aspirin	28%
Anticoagulants		
1. Should we routinely stop direct oral anticoagulants (DOACs) preoperatively?	No	8% 5/66
	Yes, for all classes	27% 18/66
	Yes, class 2 and 3	59% 39/66*
	Yes, only class 3	6% 4/66
2. When should we stop DOACs preoperatively?	Not applicable, we should not stop DOACs	0% 0/62
	48 h preoperatively if eGFR is normal	24% 15/62
	Follow trust guidance dependent on eGFR	76% 47/62***
3. When should we restart DOACs post‐operatively?	Not applicable, we should not stop DOACs	3% 2/65
	The same day	5% 3/65
	The day after surgery	83% 54/65***
	2 days after surgery	9% 6/65
Antirheumatics/DMARDs		
1. Should we routinely interrupt disease modifying antirheumatic drugs (DMARDs) perioperatively?	Yes	3% 2/70
	No	97% 68/70**
	Unsure	0% 0/70
1. If yes to question 1, how should we decide when the patient stops DMARDS?	Not applicable, we should not interrupt DMARDs	75% 48/64 ***
	Seek specialist guidance or follow local trust guidelines	25% 16/64
2. If yes to question 1, when should we restart DMARDs post‐operatively?	Not applicable, we should not interrupt DMARDs	90% 64/71 ***
	Seek specialist guidance	1% 1/71
	When wound has healed	8% 7/71
Biologics		
1. Should we interrupt biologics perioperatively?	Yes, routinely	6% 4/68
	Plan surgery at specific time during treatment cycle	59% 40/68*
	Will depend on patient's condition and risks of stopping	21% 14/68
	Seek specialist guidance	13% 9/68
	No	1% 1/68
3. Should our decision to interrupt biologics be dependent on the procedure being undertaken?	No	43% 29/68
	Yes, should interrupt for all classes	18% 12/68
	Yes, should interrupt for classes 2 and 3	38% 26/68
	Yes, should interrupt for only class 3	1% 1/68
4. When should we aim to operate on a patient receiving biologic therapy?	Mid‐cycle	3% 2/70
	End of cycle	74% 52/70**
	Follow local trust guidelines	0% 0/70
	Liaise with medical team responsible for biologic regime	23% 16/70
5. When should we consider recommencement of biologic treatment?	Not applicable, we should not interfere with regime	5% 3/65
	Next planned administration irrespective of timeframe	0% 0/65
	Seek specialist opinion	11% 7/65
	When wound has healed	85% 55/65 ***

*Note:* Key: *** High consensus > 75%, ** Moderate consensus 65%–74%, *Low consensus 55%–64%.
